# Assessment of the Dynamic Alteration of Choriocapillaris Vessel Density after Focal Laser Photocoagulation with OCT Angiography

**DOI:** 10.1155/2020/6213189

**Published:** 2020-01-24

**Authors:** Zuohuizi Yi, Yiqiao Xing, Changzheng Chen, Xiaoling Wang, Juejun Liu, Lu He, Hongmei Zheng

**Affiliations:** Eye Center, Renmin Hospital of Wuhan University, Wuhan, China

## Abstract

**Purpose:**

To evaluate the changes of choriocapillaris blood flow beneath laser lesions in noncenter-involved diabetic macular edema patients using optical coherence tomography angiography (OCTA).

**Methods:**

This was a retrospective case-series study. We used OCTA to analyze the characteristics of the choriocapillaris blood flow beneath laser lesions before laser treatment and at several intervals after treatment. The choriocapillaris vessel density (CCVD) beneath the laser lesions was based on the OCTA images and was defined as the proportion of flow pixels in the selected area calculated using FIJI software through automatic binarization processing based on threshold methods.

**Results:**

A total of 63 laser lesions in 8 eyes of 5 patients were included in this study. There was a significant decrease in the CCVD at 1 hour and 1 day following laser treatment (24.25% ± 5.04% and 22.00% ± 4.71%, respectively) when compared with the baseline value (39.09% ± 3.71%, all *p* < 0.001). The CCVD was 31.82% ± 4.53% in 1 week after laser treatment, which was significantly higher than that in 1 day after treatment (*p* < 0.001). The CCVD was 31.82% ± 4.53% in 1 week after laser treatment, which was significantly higher than that in 1 day after treatment (*p* < 0.001). The CCVD was 31.82% ± 4.53% in 1 week after laser treatment, which was significantly higher than that in 1 day after treatment (

**Conclusions:**

OCTA image analysis can reflect changes in the choriocapillaris blood flow beneath laser lesions at different times following laser treatment in vivo. Spot size and laser energy may affect blood flow recovery.

## 1. Introduction

Laser photocoagulation has been an important tool in the treatment of some retinal disorders, including retinal vein occlusion, diabetic retinopathy, and diabetic macular edema (DME). Although antivascular endothelial growth factor therapy is recommended as a first-line treatment for DME patients, focal/grid laser photocoagulation still plays a role in some specific forms of DME, such as noncenter-involved DME [1–3].

During the initial phase of laser photocoagulation, the laser-induced thermal damage is confined to the outer retina, including the photoreceptor and retinal pigment epithelium (RPE) [4]. However, previous studies conducted in both rabbits and humans have revealed that retinal morphology can be gradually repaired following laser photocoagulation [4–8].

Due to a high concentration of melanin, which absorbs a great deal of the energy, the RPE is the main target of laser photocoagulation [9]. Considering that the choriocapillaris is adjacent to the RPE and the melanin in the choroidal stroma can also absorb the laser energy, the choriocapillaris is likely to be affected by laser photocoagulation. In fact, the alterations in the choroidal microvasculature following photocoagulation have been demonstrated by several histopathologic studies [10–14]. However, to date there has not yet been a study in humans investigating the dynamic in vivo changes in the choriocapillaris following exposure to laser.

Although indocyanine green angiography (ICGA) is often used to evaluate the choroidal microcirculation, it is sometimes difficult to visualize the blood flow in the choriocapillaris due to rapid leakage of the ICG dye from the choriocapillaris that then stains Bruch's membrane and the choroidal stroma [15, 16]. Optical coherence tomography angiography (OCTA) is a new imaging technology that provides depth-resolved visualization of the retinal microvasculature and the choriocapillaris without the need for dye injection [9, 15–18]. It performs multiple repeated B-scans in the same retinal area to differentiate the moving erythrocytes in the vasculature from the static fundus structure [15, 17]. Now, the noninvasive OCTA has the capability of precisely visualizing capillary plexuses in different layers and can be utilized to assess the choriocapillaris flow changes more frequently. Recently, Cole and associates analyzed 28 laser scars from 8 DME patients with a remote photocoagulation history and classified them as deep and superficial using OCTA, specifically, that deep lesions were associated with choriocapillaris alterations while superficial ones were not [9]. However, the above study was a cross-sectional study that did not reveal if there is a dynamic change in the choriocapillaris flow.

In this study, we primarily used OCTA to assess choriocapillaris blood flow alterations at different times after focal photocoagulation in DME patients; meanwhile, we utilized other multimodel imaging, including fundus photograph and optical coherence tomography (OCT), to contrast the other corresponding changes of the retina.

## 2. Materials and Methods

### 2.1. Patients

This retrospective case-series study was conducted between February 2017 and October 2018 at the eye center of Renmin Hospital of Wuhan University. The study adhered to the tenets of the Declaration of Helsinki and was approved by the Institutional Review Board of Renmin Hospital of Wuhan University.

The eligibility criteria for this study included noncenter-involved DME with retinal thickening in the posterior pole as shown by OCT, or focal fluorescein leakage in the posterior pole as shown by fluorescein fundus angiography (FFA). Exclusion criteria were as follows: (1) patients with prior laser treatment in the posterior pole or in the macula; (2) maculopathy due to other disease; (3) retinal thickening in the posterior pole due to epiretinal membranes or vitreomacular traction; (4) patients with refraction media opacity who were unable to accept laser treatment and examination.

Prior to the laser treatment, every patient received a routine eye examination, fundus photograph, OCT, OCTA, and FFA (HRA2, Heidelberg Engineering, Heidelberg, Germany). The focal laser treatment was applied to areas with diffuse fluorescein leakage or nonperfusion as shown by FFA. The laser parameters in this study were based on the modified Early Treatment Diabetic Retinopathy Study (ETDRS) photocoagulation protocol, which included a 577 nm yellow wavelength laser (NIDEK MC-500 Vixi, Nidek, Gamagori, Japan), 0.1 second duration, 50–100 *μ*m spot size, and with 50–150 mW power in order to achieve mild or moderate intensity burns [2, 3, 19].

### 2.2. Image Acquisition

The follow-up examinations included OCTA, OCT, and fundus photograph which were performed at 1 hour, 1 day, 1 week, and 1 month after focal laser treatment. The fundus images of the posterior pole were acquired through the ResMax mode of a panoramic laser scanning ophthalmoscope (Optomap 200Tx, Optos, Marlborough, MA, USA). The en face OCTA and corresponding cross-sectional OCT images were taken by an experienced physician using an RTVue XR Avanti AngioVue imaging system (Optovue, Inc., Fremont, CA, USA). Information on blood flow was acquired using the SSADA algorithm in this device, as described in published papers [15, 16, 20]. Besides, the new 3D Projection Artifact Removal (3D PAR) technology, which can effectively reduce projection of the retinal circulation into the choriocapillaris slab, is used in this device. The 3 × 3 mm en face OCTA images consisting of laser burns in the posterior pole were captured. It is necessary to capture the images at the same position at each visit or include most of the laser burns from the last visit as much as possible (sometimes, complete consistency of the OCTA scanning location was difficult to obtain at each visit because the acquisition of the OCTA image was not in the macular fovea). The level of signal strength of the OCT and OCTA images should be more than 6/10. All images were independently reviewed and evaluated by two retinal specialists.

### 2.3. Image Analysis

In order to avoid interference from the black artifacts projected from retinal exudation or hemorrhage, the nearly circular laser spots that were clear and not covered by obvious exudation or hemorrhage were selected for analysis on the choriocapillaris layer of the OCTA images. The pixel binarization processing in the FIJI software, which is an expanded version of ImageJ version 1.51a, available at fiji.sc, free of charge, was used to analyze each laser spot. Similar algorithms were described in previous studies [21–23]. Specifically, the choriocapillaris layer of the OCTA image was first imported into FIJI. The area to be analyzed was a circular area with a diameter equal to the maximum diameter of the laser spot in the OCTA image taken one day after laser treatment and was cropped before being converted to an 8-bit image. The 8-bit image was then binarized in black and white pixels using the FIJI software command path Image > Adjust > Threshold with the IJ_IsoData method. In the binary image, the areas absent for any signal of blood flow, called flow voids, were shown in black and the areas with blood flow signal were shown in white. Lastly, the software automatically calculated the proportion of white area pixels in the selected circular area, which was defined as the choriocapillaris vessel density (CCVD) in the laser spot. Each laser spot at the different times after laser was analyzed using the same method. The circular area covering each laser spot was determined by the maximum diameter of each laser spot in the choriocapillaris layer of the OCTA image at one day after laser, as the laser spot had the largest area at that time. The region of each laser spot in the choriocapillaris layer of prelaser OCTA images was located according to the shape and distribution of the superficial retinal capillaries, and the CCVD at the corresponding area before laser was also calculated by the same method and used as the baseline value.

### 2.4. Statistical Analysis

The mean CCVD of all laser spots at different times was analyzed by one-way repeated measures ANOVA. The laser lesions formed by relatively larger spot size and higher energy (spot size ≥80 * μ*m and laser energy ≥100 mW) were classified as group A; the laser lesions formed by relatively smaller spot size and lower energy (spot size <80  *μ*m and laser energy <100 mW) were classified as group B. The baseline CCVD of the two groups before laser treatment was analyzed using the independent sample *t*-test. The CCVD change ratio one month after laser (CCVD at one month after laser/CCVD before laser) between the two groups was also analyzed using the independent sample *t*-test. All statistical analyses were performed using SPSS statistical software (version 24.0; IBM Corp, NY, USA) and *p* < 0.05 was considered significant.

## 3. Results

### 3.1. Demographic Characteristics

This study included 8 eyes of 5 DME patients meeting the inclusion criteria. Patient demographic data are shown in Table 1. Two of the five patients were male, and the mean age was 54.60 ± 6.73 years (range, 45–63).

### 3.2. Quantitative Analysis of Choriocapillaris Blood Flow Alterations beneath Laser Lesions

**Figure 1:**
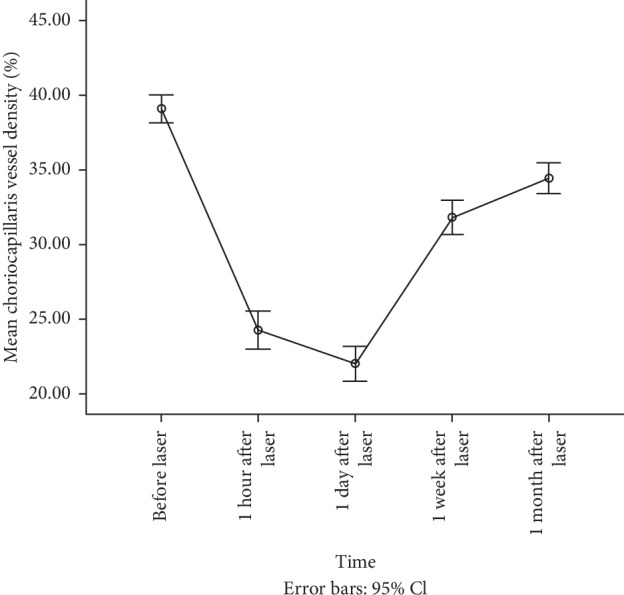
The overall changes of CCVD at different times. There was a significant decrease in the mean CCVD levels at one hour and one day after laser treatment and a significant increase at one week and one month after laser although this was a bit lower than the baseline value.

The study included a total of 63 laser spots in 8 eyes. One-way repeated measures ANOVA (Huynh–Feldt corrected) showed that there was a significant difference in the mean CCVD at different times (*F* = 290.517, *p* < 0.001). The mean CCVD of the corresponding area before laser treatment was 39.09% ± 3.71%. The mean CCVD levels at 1 hour and 1 day after laser treatment were 24.25% ± 5.04% and 22.00% ± 4.71%, respectively. These were significantly lower than the values before laser treatment (all *p* < 0.001). The mean CCVD levels at 1 week and 1 month after laser treatment were 31.82% ± 4.53% and 34.44% ± 4.16%, respectively, significantly higher than that at 1 day after laser treatment (all *p* < 0.001) (Figure 1).

**Figure 2:**
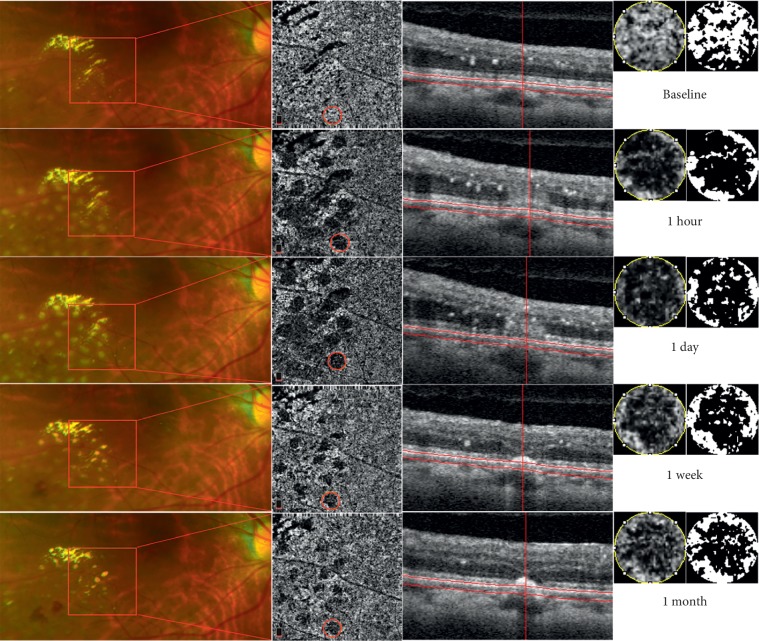
Multimodal imaging shows the laser lesions at different times in group A. The top horizontal row shows the fundus feature before laser treatment. The images from the second horizontal row to the fifth horizontal row show the laser lesions at different times, respectively. The first column is the fundus photograph taken with the Optomap® camera. The second column is the choriocapillaris layer of OCTA images shown in the red frame of the first column, which reveals an obvious decrease in the choriocapillaris flow beneath laser lesions at one hour and one day after laser treatment, whereas it recovers significantly at one week and one month after treatment. The third column is the OCT B-scan of the laser lesion circled in red in the second column, which shows a hyperreflective column-like area in the outer retinal layer at one hour and one day after laser treatment, and afterwards, the hyperreflective clumpy signal reduces to focal hyperreflective deposit overlaying RPE, with retinal edema absorption at one week and one month after treatment. The fourth column is an amplified view of the laser lesion circled in red in the second column. The fifth column is the black and white pixel image of the laser lesion after binarization.

**Figure 3:**
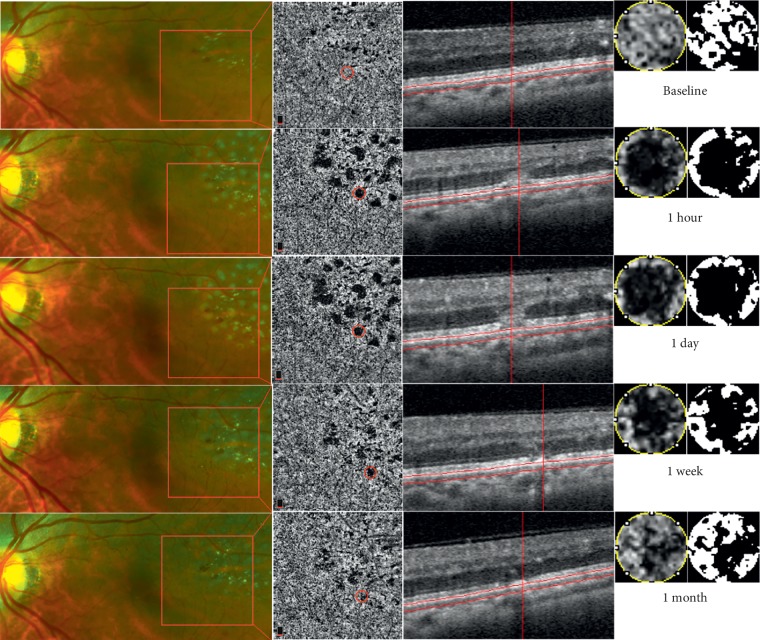
Multimodal imaging shows the laser lesions at different times in group B. The top horizontal row shows the fundus feature before laser treatment. The images from the second horizontal row to the fifth horizontal row show the laser lesions at different times, respectively. The first column is the fundus photograph taken with the Optomap® camera. The second column is the choriocapillaris layer of the OCTA images shown in the red frame of the first column, which reveals an obvious decrease in the choriocapillaris flow beneath the laser lesions at one hour and one day after laser treatment, whereas an obvious recovery is observed at one week and an almost complete recovery at one month after treatment. The third column is the OCT B-scan of the laser lesion circled in red in the second column, which shows a hyperreflective column-like area in the outer retinal layer at one hour and one day after laser treatment, and afterwards, the hyperreflective signal disappears, with a focal thickening of the RPE at one week and one month after treatment. The fourth column is the amplified view of the laser lesion circled in red in the second column. The fifth column is the black and white pixel image of the laser lesion after binarization.

Among all 63 laser spots, there were 32 laser spots in group A (Figure 2) and 31 laser spots in group B (Figure 3). The results of independent sample *t*-test analysis showed that there was no significant difference in the baseline value of CCVD between the two groups (group A: 39.48% ± 4.00% vs. group B: 38.68% ± 3.41%, *p*=0.396). The change ratio of CCVD one month after laser treatment in group A was 85.42% ± 10.46%, which was significantly lower than that of group B (91.36% ± 5.08%) (*t* = −2.877, *p*=0.006). The above results indicated a better recovery of CCVD at 1 month after laser treatment in the group with relatively smaller spot size and lower energy than for the group with relatively larger spot size and higher energy.

### 3.3. Qualitative Analysis of the Morphological Changes in the Retina following Laser

On the corresponding OCT B-scan at 1 hour and 1 day after retinal photocoagulation, each laser lesion appeared as a hyperreflective column-like area with distinct borders at the outer retinal level throughout the outer nuclear layer (ONL), the external limiting membrane (ELM), and the inner segment/outer segment (IS/OS) line. At 1 week, the hyperreflective signal at the ONL level nearly disappeared and the integrity of the ELM and IS/OS in the coagulated area was interrupted. At 1 month, each laser lesion reduced to a focal hyperreflective deposit overlaying the RPE and a focal thickening of the RPE was also detected.

The greyish-white lesion was the most obvious on the color fundus photograph one day after the laser treatment. The greyish-white lesion then became less visible at 1 week, and at 1 month, mild pigmentation was observed in the center of some laser spots.

## 4. Discussion

In the past, researchers monitoring the in vivo healing process of outer retinal layers following grid photocoagulation using OCT have found that the ELM and IS/OS progressively recovered to an intact continuous state and also observed the proliferation and migration of RPE cells to the center of the lesions over time [5, 6]. The results of our study are consistent with these findings.

To our knowledge, our study is the first to report the regularity of changes in the choriocapillaris blood flow beneath laser lesions at different times after laser treatment using OCTA in vivo. Specifically, our study revealed that there was an obvious decrease in the choriocapillaris flow in laser lesions at 1 hour and 1 day after photocoagulation, whereas there was significant recovery of the blood flow at 1 week after photocoagulation, followed by continued repair at 1 month after laser.

One of the main hypotheses for the effectiveness of laser photocoagulation is the destruction of high oxygen-consuming photoreceptors of lesioned areas and the reduction of oxygen consumption in the outer retina, thereby increasing the oxygen supply from the choriocapillaris to the inner retina through the laser scar [12, 24]. However, we found that the laser can destroy the choriocapillaris and may thus reduce the local partial pressure of choroidal oxygen and affect the diffusion of oxygen from the choriocapillaris to the inner retina [12]. Some patients complain about blurred vision, and the examination reveals an increase in retinal edema within a short period after laser, which may be related to the aggravation of transient ischemia and hypoxia caused by the effect of laser on the choriocapillaris. Fortunately, we observed that the blood flow of the choriocapillaris beneath laser spots was restored gradually over 1 week. This is also consistent with the clinical observation that patients' visual blur improves and retinal edema reduces gradually after laser. Therefore, the subsequent repair of the choriocapillaris is undoubtedly of great significance for effective laser treatment. Besides, our study found that CC perfusion at one month was further improved than that at one week, which might be partly related to the RPE atrophy resulting in an increase in the CC layer reflectivity in OCTA images, thus leading to the further increase of calculated CCVD at the last follow-ups.

As for the mechanisms of choriocapillaris damage and its subsequent restoration following retinal photocoagulation, a previous study in cats demonstrated the presence of occluded choriocapillaris containing platelet-fibrin thrombi and disruption of endothelial integrity in the photocoagulated area at 1 to 2 days after photocoagulation. At 4 days, the platelet-fibrin thrombi within the choriocapillaris were absent and the appearance of regenerative endothelia was observed. At 10 to 20 days, the structures of the choriocapillaris were gradually restored. At 30 days, the endothelia fenestrae adjacent to Bruch's membrane were clearly present and the damaged choriocapillaris was almost repaired completely [10]. These results in cats were consistent with the change rule of the choriocapillaris blood flow of the laser lesions in our present study, and thus we supposed that in the human eye, restoration of the choriocapillaris following laser treatment may also be due to thrombolysis and endothelia activation. As for the lowest CCVD level that occurred 1 day after laser treatment, rather than 1 hour after, this is consistent with the time point of thrombosis.

Based on previous research [9, 11–13], it was determined that the possible factors influencing the recovery of the choriocapillaris flow after laser included the size of the laser spot and the level of energy intensity of laser photocoagulation. Lee and associates observed three sizes of laser lesions in cats (larger than 1 mm, 500  *μ*m, and 200  *μ*m) for changes in choroidal circulation and found that choriocapillaris loss is more evident in larger lesions than in smaller ones at 4 to 5 weeks after laser photocoagulation [12]. It was consistent with our study, which suggested that the larger the lesion, the worse the repair. Furthermore, a previous study in cats regarding the different levels of energy intensity revealed that obstruction of the choriocapillaris was initially present in all lesions 24 hours after laser and that a larger diameter of choriocapillaris defect in the photocoagulated area accompanied higher laser intensity. At 30 days, the choriocapillaris defects produced with threshold and moderate intensity energy appeared almost completely refilled, whereas defects created by high-intensity energy showed incomplete refilling [11]. Because different structures could absorb laser energy differently, the severity of choriocapillaris defects might be, to some degree, different between cats and humans [12]. However, in the present study, we found that small and mild laser lesions could be repaired completely, while large and intense lesions would result in incomplete repair, which was consistent with animal research. Thus, the optimization of laser parameters and formulation of individual laser therapy regimen would be necessary in order to achieve the best therapeutic effect and minimize the effect on the choriocapillaris blood flow as much as possible.

Our study does have several limitations. First, the sample size was small and the subgroup analysis could be more detailed. There was no graded comparison based on the different levels of laser energy and spot size. Second, our study was a retrospective study. Third, although the literature [25] reports that some light laser lesions might be stable in 30 days, the follow-up time was short. Last, though the binarization results can reflect the choriocapillaris blood flow to some extent, it is nevertheless, not the true vessel density. We use the definition of CCVD to describe the blood flow changes expediently.

In conclusion, OCTA can be used to observe the changes of the choriocapillaris blood flow beneath laser lesions at different time points after photocoagulation in vivo. Our results showed that after laser treatment, the choriocapillaris blood flow beneath the laser lesions decreased significantly at 1 hour and 1 day, with an obvious recovery at 1 week and 1 month after laser. Furthermore, near-complete recovery was achieved in the choriocapillaris beneath some laser spots, while others only displayed partial recovery at 1 month. The final recovery may be related to the laser spot size and laser energy. In the future, the impact of different laser modes, for example, the comparison of Pattern Scan Laser (PASCAL), traditional single-spot laser, and micropulse laser on choriocapillaris blood flow should be further explored.

## Figures and Tables

**Table 1:** Demographic data of the patients.

Patient	Gender	Age (years)	Eye	Spot size (*μ*m)	Laser power (mW)	Numbers of laser spots	Group
1	Female	63	od	100	150	7	A
			os	50	70	8	B
2	Male	52	od	100	140	8	A
			os	90	120	8	A
3	Female	58	od	80	120	9	A
4	Female	55	od	70	70	7	B
			os	60	80	8	B
5	Male	45	od	50	70	8	B

od, oculus dexter (latin), right eye; os, oculus sinister (latin), left eye.

## Data Availability

The data supporting the results of the current article are available from the corresponding author upon request.
